# The Role of Single Nucleotide Polymorphisms in MicroRNA Genes in Head and Neck Squamous Cell Carcinomas: Susceptibility and Prognosis

**DOI:** 10.3390/genes15091226

**Published:** 2024-09-20

**Authors:** Elżbieta Szmida, Dorota Butkiewicz, Paweł Karpiński, Tomasz Rutkowski, Małgorzata Oczko-Wojciechowska, Maria Małgorzata Sąsiadek

**Affiliations:** 1 Department of Genetics, Wroclaw Medical University, 50-367 Wroclaw, Poland; elzbieta.szmida@umw.edu.pl (E.S.); maria.sasiadek@umw.edu.pl (M.M.S.); 2 Center for Translational Research and Molecular Biology of Cancer, Maria Skłodowska-Curie National Research Institute of Oncology Gliwice Branch, 44-102 Gliwice, Poland; dorota.butkiewicz@gliwice.nio.gov.pl; 3 Radiotherapy Department, Maria Skłodowska-Curie National Research Institute of Oncology Gliwice Branch, 44-102 Gliwice, Poland; tomasz.rutkowski@io.gliwice.pl; 4 Department of Clinical and Molecular Genetics, Maria Skłodowska-Curie National Research Institute of Oncology Gliwice Branch, 44-102 Gliwice, Poland; malgorzata.oczko-wojciechowska@gliwice.nio.gov.pl

**Keywords:** head and neck cancer, HNSCC, miRNA, polymorphisms, cancer risk, prognosis, biomarkers, SNPs

## Abstract

Background: Head and neck squamous cell carcinoma (HNSCC) is one of the most prevalent cancers worldwide. The identification of molecular alterations adding to the individual risk of HNSCC development and progression is one of the most important challenges in studies on cancer genetics. MicroRNAs (miRNAs), which belong to the group of important post-transcriptional regulators of human gene expression, seem to be valuable options for consideration as key modifiers of individual cancer risk, and therefore may be helpful in predicting inter-individual differences in cancer risk, response to treatment and prognosis. Methods: There have not been many studies focused on the relationship between miRNA variants and HNSCC published in PubMed within the last 15 years. We found and analyzed 30 reviews, meta-analyses and research papers and revealed 14 SNPs which have been reported as significant in the context of HNSCC susceptibility and/or prognosis. Results: These 14 SNPs were located in 13 separate miRNAs. Among them, four were the most frequently studied (miRNA-146, -196, -149 and -499) and have been shown to have the greatest impact on the course of HNSCC. However, the presented results have been conflicting. Conclusions: It must be concluded that, despite the years of studies, there are no conclusive reports demonstrating a significant role of SNPs in miRNAs in the context of the susceptibility to HNSCC or its prognosis.

## 1. Introduction

Head and neck squamous cell carcinoma (HNSCC) is one of the most prevalent cancers worldwide. According to GLOBOCAN, in 2020, HNSCC accounted for about 5% of all cancer cases and caused about 4.7% of cancer-related deaths [1]. Nearly 90% of HNSCCs originate from the mucosal surface of the oral cavity, pharynx or larynx [2,3]. The most common risk factors for HNSCC are tobacco use and alcohol consumption; however, a significant increase in HNSCC cases in recent decades is associated with human papillomavirus (HPV) infection (especially HPV type 16), which is related to oropharyngeal cancer. Further, Epstein–Barr virus (EBV) infection is associated with nasopharyngeal carcinoma (NPC) [4,5]. In early-stage HNSCC, the standard treatment includes surgery or radiotherapy as separate procedures or in combination. In the advanced stages, surgery, radiotherapy and chemotherapy are usually combined [6,7]. The prognosis for patients suffering from HNSCC depends mostly on the clinical stage of the disease, and locoregional recurrence is the most common cause of an unfavorable course of the disease [8]. In patients with recurrent or metastatic HNSCC, the results of treatment are poor, and the median survival time is about 12 months [9]. Despite immense progress in diagnosis and therapy, the 5-year survival rate of HNSCC patients has not improved significantly [10]. Therefore, there is a great need for new, effective biomarkers for early diagnosis, treatment and prognosis.

MicroRNAs (miRNAs) are short, 18-25 nucleotide single-stranded non-coding RNAs that regulate gene expression by inhibiting the translation of targeted genes, silencing messenger RNAs (mRNAs) and accelerating their degradation [11]. miRNAs recognize targets based on specific base-pairing complementarity between the seed sequences of miRNAs (5′ end) and 3′ or 5′ untranslated regions (UTRs) of target genes/mRNAs. Thus, miRNAs play a vital role in many biological processes, like cell proliferation, differentiation, migration, angiogenesis, apoptosis and cell-to-cell communication [12,13]. Genome-wide miRNA expression profiling has demonstrated that almost all cancer types show specific profiles of up- and down-regulated miRNAs [14,15]. Dysregulation of mature miRNAs expression plays a causal role in cancer development and progression. They have been identified to function either as tumor suppressors or promoters. miRNAs are characterized by high stability, making them good biomarkers for detection, not only in solid tissue samples, but also in serum or urine [16]. Single nucleotide polymorphisms (SNPs) represent the most prevalent type of variation in the human genome.

miRNA SNPs may modulate miRNA function by changing the transcription of the primary transcript, processing pri-miRNAs and pre-miRNAs and affecting miRNA–mRNA interactions. SNPs may be located in different regions of miRNA genes: in the precursor sequence, in the promoter region or in the seed sequence. SNPs in the miRNA precursor region potentially modulate the expression and processing of miRNAs. SNPs in promoter regions affect the expression of mature miRNAs, whereas SNPs in the seed sequence may impact the binding of the miRNA to the target mRNA [17,18]. Single nucleotide changes in the seed region or shifts in the processing sites during the biogenesis of the miRNA/miRNA* duplex may result in a new miRNA molecule with new target genes [19]. SNPs in miRNAs can also alter target recognition (target-binding affinity and specificity), which may have diverse functional consequences, such as changes in the targeted gene expression or induction of aberrant maturation. Single nucleotide changes in miRNA may alter the influence of a given miRNA on a variety of processes, such as cell proliferation, differentiation, migration, angiogenesis, apoptosis or cell-to-cell communication, thus influencing individual cancer susceptibility as well as the clinical outcomes of the disease.

Many SNPs have been extensively studied as potential biomarkers of cancer risk, therapy response and prognosis in various malignancies. miRNA-related SNPs may influence gene expression, cause disturbances in miRNA biogenesis and lead to alterations in target recognition. It has been shown in recent epidemiological studies on different populations that some SNPs in miRNA genes contribute to HNSCC susceptibility and clinical outcomes, as well as responses to treatment [20,21,22]. Thus, research on genetic variation in miRNAs may be particularly important for understanding the biology of HNSCC tumors, developing new diagnostic strategies and identifying more effective therapies.

## 2. Results of the Analysis

### 2.1. SNPs in miRNA

A number of gene expression studies have highlighted that miRNAs may have the potential to be used as prognostic biomarkers in HNSCC patients. However, the studies on the role of miRNA SNPs in HNSCC which have been published so far have yielded inconsistent results.

**Figure 1:**
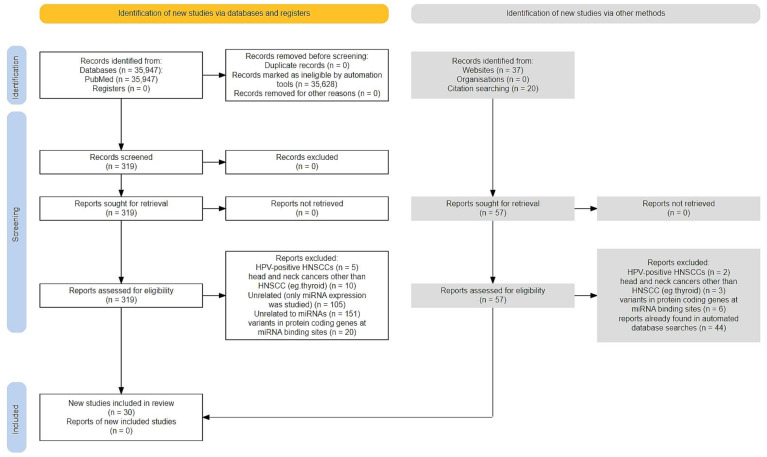
Selection of studies of associations between genetic variants in miRNAs and HNSCC, created in the PRISMA2020 Shiny R package [23].

Therefore, we aimed to summarize the current data on the associations between miRNA SNPs and HNSCC risk and prognosis. We gathered data from the NCBI PubMed database using a text mining method (R version 4.2.2/pubmedR 0.0.4) and manual browsing of abstracts which were published within the last 15 years (2008–2022) (see flowchart provided in Figure 1). Our search resulted in a collection of 35,974 abstracts. Titles and abstracts of the retrieved articles were then analyzed to exclude items concerning HPV-positive HNSCCs (because of their different biology and genetics), head and neck cancers other than HNSCC and thyroid cancer, as well as papers concerning SNPs located at miRNA binding sites and miRNA expression. The final list contained 30 reviews and meta-analyses, as well as research articles. None of the meta-analyses were derived from bioinformatic re-analysis of publicly available SNP array data.

**Figure 2:**
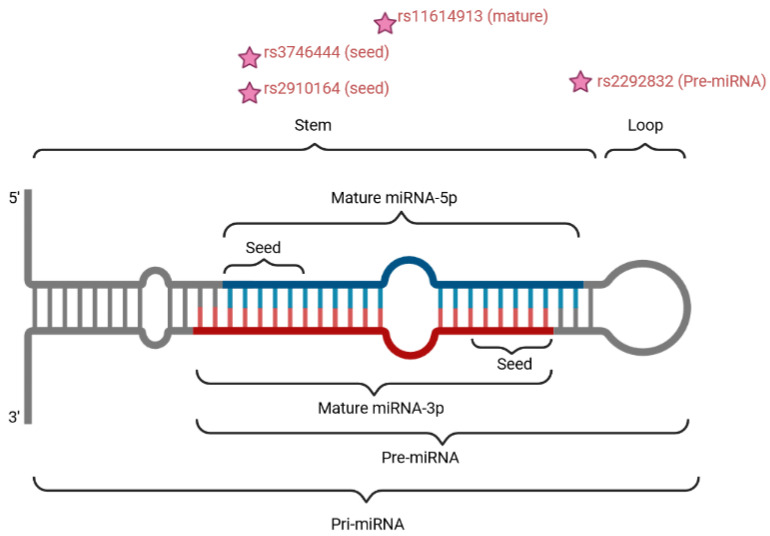
Localization of the four most common SNPs with respect to miRNA conformation. Precursor miRNA sequence hairpin conformation with indicated positions (stars) of the four most common SNPs studied in the context of HNSCC. Created with BioRender.com.

The most frequently studied SNPs in the selected set of papers were miR-146a rs2910164, miR-196a-2 rs11614913, miR-149 rs2292832 and miR-499 rs3746444 (Figure 2, Table 1, Supplementary Table S1). SNPs that were rarely described in the literature are presented in Table 2.

### 2.2. MiRNA-146a and rs2910164

Together with miR-146b, miR-146a belongs to the microRNA-146 family. The genes are located on two different chromosomes (5 and 10, respectively), and are not transcribed in tandem. Their transcription is regulated by different factors and co-factors, and they also display different tissue specificity. Despite all these differences, they have very similar sequences and share common seed regions [53].

Alterations in miR-146a expression are common phenomena in immune responses, inflammation, apoptosis and tumorigenesis, as miR-146a is involved in the regulation of a variety of different pathways [54,55,56,57,58]. Moreover, miR-146a is known for its antagonistic role in inflammatory processes and for repressing the immune response by negative regulation of toll-like receptors (TLRs) and NFkB signaling pathways. These pathways are activated by the ligands of the toll-like receptors, such as tumor necrosis factor α (TNFα) and interleukin-1β (IL-1 β) [59]. The relationship described above—the regulatory loop—has been suggested to be an important causal link between inflammation, carcinogenesis and differentiation [60,61].

The rs2910164 SNP was first described in papillary thyroid carcinoma [62]. It is located on the passenger strand of pri-miR-146a. The G to C substitution results in the formation of three types of mature miR-146 via the process of different cutting by a microprocessor from the leading strand (miR-146a) and from the passenger strand (miR-146a*G and miR-146a*C). Cleavage at the apical junction destroys the miRNA sequence by altering its processing due to the G:U to C:U exchange in the stem region, thus leading to a base mismatch and reduction in miR-146a expression [63,64]. In the dbSNP database, this SNP is reported as a C>G substitution. The role of rs2910164 in HNSCC development and progression has been presented in 17 papers published in PubMed within the last 15 years (see Table 1) [20,24,25,26,27,28,29,30,31,32,33].

Due to the differences in the rs2910164 allele frequency in different populations, some authors considered G (G>C) to be the major allele, while others considered it to be C (C>G) (Table 1). This makes the interpretation of the results challenging. However, the G>C polymorphism is more frequently examined in the selected papers. A predisposition to HNSCC in carriers of the C allele in the miR-146a precursor [30] has been reported in six studies. Most of the authors suggested an association between the C allele (GC and CC genotypes) and an increased risk of HNSCC (HNSCC subtypes and NPC) in Asian and Caucasian populations [20,24,25,26,31].

Contradictory results have been shown in three other papers in which the G allele has been linked to an increased risk of HNSCC in the Asian population (Chinese population, dominant model) [27], Asian and Caucasian populations in a dominant model after analysis stratified by ethnicity in Chinese and Taiwanese populations (homozygote and dominant model) [29], as well as in Chinese males with oral squamous cell carcinoma (OSCC) (however, after Bonferroni correction, the association was statistically insignificant) [32]. A correlation between at least one G allele (C>G), OSCC risk and environmental factors like betel and tobacco use has been found in a Taiwanese population [34]. Zeng et al. showed that the CC (G>C) genotype had a protective effect against OSCC in Chinese, Taiwanese and Italian populations [28], while Chen et al. found that the GG (C>G) genotype reduced the risk of oral premalignant lesions, oral cancer and pharyngeal cancer in a Taiwanese population [35]. There were also five reports showing no association between rs2910164 SNPs and HNSCC risk in Caucasian as well as Taiwanese populations [34,36,37,38]. Wang et al. showed no effect of the SNP on clinical outcome (overall survival—OS; disease specific survival—DSS; and disease free survival—DFS) in non-Hispanic white patients with SCCNOP (squamous cell carcinoma of the nasopharynx) [39]. However, Chen et al. observed an association between the G>C polymorphism and disease-free survival (DFS) in a non-Hispanic white population with OPSCC (oropharynx squamous cell carcinoma) [33].

### 2.3. MiR-196a-2 and rs11614913

miR-196a-2, together with miR-196a-1 and miR-196b, belong to the microRNA-196 family. These miR-196 genes are located on three different chromosomes (12, 17 and 7, respectively) in the regions of homeobox (*HOX*) clusters. miR-196a-1 and miR-196a-2 genes are transcribed into the same functional mature miRNA [65]. The rs11614913 SNP is located on the mature sequence of miR-196a*, which is one of two products of the maturation of miR-196a-2 (the second being miR-196a). Both of them are processed from the same stem-loop. The presence of C to T substitution has a negative impact on the formation of a mature miRNA, expression of target genes (including many cancer-related genes, such as the *HOX* gene family or *TP63*) and on the cell cycle [66]. According to the dbSNP database, there are two possible variants: C>G and C>T.

We found 13 papers concerning the role of rs11614913 in HNSCC. Five of them were meta-analyses [20,25,28,31,40] while eight were research and case-control studies [32,33,34,36,37,39,41,42] (Table 1). These studies were conducted mainly on Asian or Caucasian populations, and in the majority of the studies, the C allele was considered to be the major allele (C>T). In just one study, the T allele was identified as the predominant allele [32]. Miao [36] et al. also took into the consideration the A>G polymorphism, but such an rs11614913 variant has not been reported in the dbSNP database. Therefore, the authors of the meta-analyses considered a T>C instead of A>G exchange. The same assumption has been made for the purposes of this mini review. The rs11614913 T variant (C>T) has been shown to be associated with an increased risk of HNSCC, particularly in the Asian population [20,31,32,40,41]. Contradictory results have been presented by Christensen [42] et al., as their study suggests that the T (C>T) allele is associated with a reduced risk of HNSCC in Caucasian patients. Two meta-analyses and three case-control studies showed no association between the rs11614913 SNP and the risk of HNSCC in both Asian and Caucasian populations [25,28,32,34,37]. Moreover, several studies on the association between the rs11614913 SNP and HNSCC patients’ survival rates showed contradictory results. Li et al. observed that the rs11614913 T (C>T) allele may contribute to NPC risk, as well as progression in NPC patients, since this variant was associated with local tumor invasion and advanced lymph node metastasis [41]. Chen et al. revealed that OPSCC patients with a CC (C>T) genotype had significantly worse DFS and increased recurrence risk compared to those with the variant genotypes TC and TT [65]. Christensen et al. observed that pharyngeal cancer patients with TT homozygotes had significantly reduced survival time, suggesting that the protective effect of the variant was not related to disease severity [42]. On the contrary, Wang et al. did not observe a relationship between this SNP and the clinical outcome (OS, DSS and DFS) of disease or the survival time of patients with SCCNOP [39].

### 2.4. MiR-149 and rs2292832

miR-149 is encoded by one exon of the *MicroRNA-149* gene located on chromosome 2q37.3. The miR-149 hairpin products are miR-149-5p (guide strand) and miR-149-3p (passenger strand). Both transcripts have different sequences, suggesting their different biological functions. This has been confirmed in studies showing that both transcripts may act as an oncogene and/or tumor suppressor. However, each of them targets a different pathway and different genes; for example, miR-149-5p plays a role in the ERBB, insulin, MAPK and chemokine signaling pathways, while miR-149-p3 participates in toll-like receptor, T and B cell receptor, focal adhesion, vascular smooth muscle contraction and lysosome pathways. Thus, miR-149 may affect key processes in cancer proliferation, apoptosis, migration and invasion [67,68]. Moreover, Shen et al. linked low expression of miR-149-3p with malignant development and poor outcomes in patients with OSCC [69].

The rs2292832 polymorphism is located in the precursor region of miR-149 and does not reside in the mature sequences of either miR-149-5p or miR-149-3p. Nevertheless, the presence of the T variant in the pri-mir-149 sequence results in low conversion efficiency of pre-mir-149 to miR-149 [70]. The presence of this SNP results in the alteration of mature miRNA expression and binding mechanisms to target mRNA [43]. According to the dbSNP database, T>A and T>C are possible variants.

We found nine reports concerning the role of rs2292832 in HNSCC. Three of them were meta-analyses [20,25,28] and six were research or case-control studies [33,34,36,37,39,43] (Table 1). The studies were conducted mainly in Asian or Caucasian populations. According to the dbSNP database, the rs2292832 has two variant alleles T>A and T>C, but most of the authors took C as the major allele and T as the minor allele (C>T). In two out of the nine analyzed reports, the authors took into account A>G and G>A allele variants which are not listed in the dbSNP database. However, in the literature the A>G and G>A were considered as the T>C and C>T, so we also assumed that in our mini review. Most of the studies show no association of this SNP with HNSCC susceptibility [20,25,28,36,37,39] or with OS, DSS or DFS [33]. However, it has been observed in the population of Taiwan that at least one C allele (genotypes CC, TC; T>C) correlated with an increased risk of OSCC in betel nut or/and tobacco consumers, suggesting a strong impact of this SNP on oral cancer susceptibility [34]. In turn, the study by Tandon et al. revealed a significant association between the T allele (genotypes TT, CT; C>T) and susceptibility to OSCC in the Indian population [43]. Wang et al. reported significantly lower risk of death and disease recurrence in SCCNOP patients with CC (C>T polymorphism) who had been smokers ever in their lifetime and were treated with radiation and/or chemotherapy with or without surgery [39].

### 2.5. MiR-499 and rs3746444

The miR-499 gene is located on chromosome 20q11.22. Wang et al. reported that miR-499 regulates mitochondrial dynamics by targeting calcineurin and dynamin-related protein-1, resulting in their suppression via p53, which permits the execution of an apoptotic pathway [71]. MiR-499 directly targets proteins such as forkhead box O4 (FOXO4) and programmed cell death 4 (PDCD4). Therefore, miR-499 may promote cancer migration and invasion in colorectal cancer patients via modulation of PDCD4 activity [72], while miR-499 plays a key role in numerous important biological processes, such as modulation of the immune response, cell proliferation, apoptosis, neuromuscular regulation, neoangiogenesis and inflammation [43,73]. The rs3746444 A>G SNP is located in the seed region of miR-499. The seed region is essential for miRNA-mediated gene silencing and may influence both target binding ability and maturation processes, which in turn affect susceptibility to human diseases including cancer [43,74]. The presence of rs3746444 within the stem region of the miR-499 gene results in an A:U to G:U mismatch in the stem structure of the miR-499 precursor, which affects binding to genes related to the process of carcinogenesis, such as Sox6 and Rod1 [25].

We present the results of the analysis of 12 papers concerning the role of rs3746444 in HNSCC. Four of them were meta-analyses [20,25,28,31] and eight were research or case-control studies [32,33,34,37,39,43,44,45] (Table 1). According to the dbSNP database, rs3746444 has two possible variants: A>C and A>G. Some authors took into consideration the T>G variant, which is not listed in the dbSNP database, so we considered this variant as an A>C. These studies were conducted in either Asian or Caucasian populations. Most authors show that the presence of at least one G allele increases the risk of HNSCC in the Asian population, as analyzed by different statistical models [28,32,34,43]. Contradictory results were obtained in a study of the Taiwanese population, as it has been suggested that the G (A>G) allele contributes to increased risk of betel quid-related oral submucous fibrosis (OSF), along with decreased risk of OSCC. These differences could be caused by the presence of the rs3746444 G variant which influences the expression of miR-499a-5p in tumorigenesis of OSCC [44]. Reduction of HNSCC and NPC risk associated with the presence of at least one G allele was observed in Caucasian populations in two studies [31,37]. However, other authors found no similar association in either Caucasian or Asian populations [20,25,33,44]. The study of Wang et al. showed significantly reduced risk of overall death, disease specific death and risk of recurrence in AA genotype carriers who had been smokers at any point in their lives and were treated with radiation and/or chemotherapy with or without surgery [39].

### 2.6. Other SNPs

Further, there are a few other SNPs, such as miR-608, miR-605 or miR-34b, potentially associated with HNSCC risk, which were described in only one or two studies (Table 2) [34,36,46,47,48,49,50,51,52]. The results of these studies revealed a significant relationship between these polymorphisms and HNSCC or OPL (oral premalignant lesion). Nevertheless, due to the insufficient number of reports, an assessment of these variants is not possible. Further research in large, ethnically homogenous groups is necessary to validate these observations.

### 2.7. SNP Combinations

Several studies consider the influence of SNP combinations on HNSCC risk. We found four research articles [33,36,37,48,51] and one case-control study concerning SNP combinations and HNSCC risk [36]. Miao et al. showed that the co-presence of miR-605 rs2043556 and miR-196a2 rs11614913 was significantly associated with HNSCC risk in a Chinese population in a dose-response manner. The comparison between the groups with 0–2 and 3–4 risk alleles showed the risk of OSCC increasing significantly with the number of adverse alleles [36]. In another study on Chinese OSCC patients, a significant locus–dose relationship, increasing with the number of unfavorable alleles, was found between miR-101 rs578481 and rs705509 and increased risk of OSCC [48]. In a combined analysis of four SNPs (miR-146a rs2910164, miR-149 rs2292832, miR-196a2 rs11614913 and miR-499 rs3746444), a relationship between the number of adverse genotypes and a moderately increased risk of HNSCC was found in a non-Hispanic white population [37]. Roy et al. studied SNPs in two miRNAs (miR-196-a2 rs11614913 and miR-34-b rs2187473) and two miRNA processing genes (Ran rs14035 and Gemin3 rs197412) in an Indian population and showed a significantly higher risk of oral cancer in carriers of 4–6 (1.8-fold increase) and 6–8 risk alleles (3-fold increase) [51]. In a combined risk analysis including nine polymorphisms (miRNA-146 rs2910164, miRNA-196 rs11614913, miRNA-149 rs2292832, miRNA-499 rs3746444, miRNA-492 rs2289030, miRNA-423 rs6505162, Gemin4 rs910924, Gemin3 rs197388 and TRBP rs784567), Chen et al. found that, based on the number of unfavorable alleles, their Caucasian population could be stratified into a low-risk reference group with less than four risk genotypes; a medium-risk group with 4–5 risk genotypes (a 1.7-fold increase of the risk); and a high-risk group with more than five risk-related genotypes, which is characterized by a threefold increase in the risk of SCCNOP. Chen et al. also reported an association between these combined polymorphisms and the risk of recurrence, showing an increase in risk with an increased number of adverse alleles [33]. As the above-mentioned studies suggest, there is a strong correlation between the number of risk-related alleles and HNSCC susceptibility. A combined analysis of SNPs together with other risk factors, such as smoking and alcohol consumption, as well as environmental exposure, may better characterize the high-risk groups.

## 3. miRNA Variants in Other Squamous Cancer Types

Using a database of scientific abstracts from the years 2008–2022, 55 abstracts were identified that relate to studies on miRNA gene variants and the risk of squamous cell carcinomas (excluding HNSCC). Of these, 62% (*n* = 34) pertained to esophageal squamous cell carcinoma, 21% (*n* = 12) to cervical cancer and 16% (*n* = 9) to lung cancer. Similarly to the approach described above; in order to identify significant associations between miRNA variants and cancer risk, we focused on variants that were commonly examined (in at least two independent studies). The most frequently studied variants in the context of ESCC were rs11614913 (miR-196a-2) and rs2910164 (miR-146a), which we have previously discussed in relation to HNSCC. Each of these variants has been described in seven separate studies [75,76,77,78,79,80,81]. A meta-analysis published in 2018 by Guo et al., analyzing 13 studies (the study included over 3000 ESCC cases and over 4000 controls), demonstrated in a pooled analysis that individuals in a Chinese population with the TT variant genotype of rs11614913 in the miR-196a-2 gene had a significantly decreased risk of ESCC compared to those with the CC genotype [82]. This association was also shown for the allelic model. No significant associations were observed for rs2910164. The most recent study, published in 2021 (encompassing 829 cases and 1522 controls), found no significant associations between rs11614913 and rs2910164 with the risk of ESCC in a Chinese population [75]. The next two most frequently studied variants, rs4938723 (pri-miR-34b/c) and rs6505162 (miRNA-423), were investigated in five studies (including over 2000 ESCC cases and over 2000 controls). A significantly reduced risk of ESCC was observed in a pooled analysis of rs4938723 (CC genotype vs. TT), but no significant association was found for rs6505162 [82]. The least frequently studied variant, examined in three publications, was rs3746444 (miR-499), which has also been discussed in this study in the context of HNSCC [75,76,79]. Liu et al. found a statistically significant association between the risk of ESCC and the rs3746444 GG genotype (829 cases and 1522 controls) [75], confirming earlier findings by Shen et al. (with 1400 ESCC cases and 2185 matched controls) [76].

Regarding the other cancer types for which miRNA association studies were identified (cervical and lung cancer), all the variants found were examined in only a single study. Therefore, we decided not to include them in this paper. In summary, studies on miRNA variants in the context of squamous cell carcinomas other than HNSCC are still in their early stages and are limited to a few cancer types, with ESCC being the most frequently studied. Furthermore, it should be emphasized that all the cited studies in this context were conducted in Asian populations.

## 4. Conclusions

The published data suggest that various miRNA gene variants may modify the risk of HNSCC development and progression, as well as the response to therapy and prognosis. However, these relationships have not been fully explored, and existing results are often inconsistent. In addition, the published data concern a very limited number of miRNA polymorphisms.

In the present review, we have summarized the data available so far regarding SNPs located in selected miRNAs and their associations with individual cancer risk and the clinical course of the disease in HPV-negative HNSCC. During our literature search, we identified 14 SNPs in 13 miRNAs that have been associated with altered cancer risk, response to treatment and prognosis. Among them, four were the most frequently studied (miRNA-146 rs2910164, -196 rs11614913, -149 rs2292832 and -499 rs3746444). The data we have collected from the literature did not provide a clear answer as to the significance of the SNPs in the miRNAs described above. There are several possible causes of these discrepancies. Firstly, in many reports, the study groups were not precisely stratified according to important determinants of HNSCC risk, such as HPV-positive and -negative status, location of primary tumors, lifestyle differences and environmental exposure. These variables have not been taken into account. In recent years, epidemiological studies have shown that in the pathophysiology of many diseases, including cancers, the interactions of environmental factors with specific allelic variants noticeably modulate susceptibility to diseases [22]. Most of the studies into this matter have involved Asian or Caucasian cohorts, therefore more research on the role of miRNA SNPs in different populations is needed to determine if some genetic variants are specific only to a particular population/ethnicity or if they are, in general, common for all subjects. Moreover, a simultaneous evaluation of a panel of miRNA-related polymorphisms is more likely to identify genetic biomarkers of risk than the assessment of a variation at a single locus.

Considering the important role of miRNAs in gene regulation and their significance in the development and course of human diseases, including cancer, further studies are warranted. Research should be conducted in large groups, homogenous in terms of lesion location and ethnicity.

## Figures and Tables

**Table 1:** Most common polymorphisms in miRNAs in HNSCC.

rs	miR	Allele/Genotype	Case/Controls	Role in HNSCC	Type of Cancer	Population/ Ethnicity	References
**rs2910164**	**146a**	**C (G>C)**	**3841/7900**	**increased risk—Caucasian population**	**HNSCC**	**Caucasian, Asian**	**[20]**
**rs2910164**	**146a**	**C (G>C)**	**2485/11034**	**increased risk—HNSCC; laryngeal-dominant and homozygote model; nasopharyngeal-homozygote model**	**HNSCC**	**Asia (Chinese)**	**[24]**
**rs2910164**	**146a**	**C (G>C)**	**1577/1598**	**increased risk—heterozygote comparison**	**HNSCC**	**Caucasian (USA, Hungarians), Asian (China)**	**[25]**
**rs2910164**	**146a**	**C (G>C)**	**389/3813**	**increased risk**	**NPC**	**Asia (Chinese)**	**[26]**
**rs2910164**	**146a**	**GG, GC (C>G)**	**2766/6603**	**increased risk—Chinese dominant model**	**HNSCC**	**Caucasian (Hungarians, American), Asia (Chinese)**	**[27]**
**rs2910164**	**146a**	**CC (G>C)**	**2323/5059**	**protective**	**OSCC**	**Asia (Chinese, Taiwan), Caucasian (Italy)**	**[28]**
**rs2910164**	**146a**	**G (C>G)**	**3634/7818**	**increased risk—dominant model; stratified analysis by ethnicity; Asians—homozygote and dominant model**	**HNSCC**	**Caucasian (USA, Italy, Hungary), Asian (China, Taiwan)**	**[29]**
**rs2910164**	**146a**	**C (G>C)**	**4412/8781**	**increased risk**	**HNSCC**	**Caucasian, Asian**	**[30]**
**rs2910164**	**146a**	**C (G>C)**	**393/373**	**increased risk (susceptibility)**	**NPC**	**Caucasian, Asian**	**[31]**
rs2910164	146a	G (C>G)	340/340	increased risk in males; the results were not statistically significant after Bonferroni correction	OSCC	Asia (Chinese)	[32]
rs2910164	146a	GG (G>C)	1008/-	reduced risk of recurrence	OPSCC	Caucasian (Non-Hispanic white)	[33]
rs2910164	146a	(C>G)	470/425	no association; increased risk with betel nut chewing and tobacco use	OSCC	Asia (Taiwan)	[34]
rs2910164	146a	GG (C>G)	658/668	reduced risk	OSCC, PSCC, OPSCC	Asia (Taiwan)	[35]
rs2910164	146a	(G>C)	576/1552	no association	HNSCC	Asia (Chinese)	[36]
rs2910164	146a	(G>C)	1109/1130	no association	HNSCC	Caucasian (Non-Hispanic white)	[37]
rs2910164	146a	(G>C)	346/population from HapMap project (TSI)	no association	OSCC	Caucasian (Italian)	[38]
rs2910164	146a	(G>C)	996/-	no association	SCCNOP	Caucasian (Non-Hispanic white)	[39]
**rs11614913**	**196a2**	** T (C>T)**	**3534/3564**	**increased risk—Asian population**	**HNSCC**	**Caucasian, Asian**	**[20]**
**rs11614913**	**196a2**	** (C>T)**	**1593/1685**	**no association**	**HNSCC**	**Caucasian, Asian**	**[25]**
**rs11614913**	**196a2**	** (C>T)**	**2138/2557**	**no association**	**OSCC**	**Asia**	**[28]**
**rs11614913**	**196a2**	**T (C>T)**	**5189/5213-HNSCC;** **1561/1304-OSCC**	**increased risk—co-dominant model CC vs. CT-Asian population; OSCC susceptibility**	**HNSCC**	**Caucasian, Asian**	**[31]**
rs11614913	196a2	(T>C)	340/340	no association	OSCC	Asia (Chinese)	[32]
rs11614913	196a2	CC (C>T)	1008/-	increased recurrence risk	OPSCC	Caucasian (Non-Hispanic white)	[33]
rs11614913	196a2	C (T>C)	470/425	no association; increased risk with betel nut chewing and tobacco use	OSCC	Asia (Taiwan)	[34]
rs11614913	196a2	G (A>G)	576/1552	increased risk—OSCC	HNSCC	Asia (Chinese)	[36]
rs11614913	196a2	(C>T)	1109/1130	no association	HNSCC	Caucasian (Non-Hispanic white)	[37]
rs11614913	196a2	(C>T)	996/-	no association	SCCNOP	Caucasian (Non-Hispanic white)	[39]
**rs11614913**	**196a2**	**TT, CT (C>T)**	**3534/3564**	**increased risk (susceptibility)**	**HNSCC**	**Caucasian, Asian**	**[40]**
rs11614913	196a2	CT, TT (C>T)	1084/1036	increased risk (susceptibility); TT genotype had a significantly increased frequency of invasion of local tumor and advanced lymph node metastasis	NPC	Asia (Chinese)	[41]
rs11614913	196a2	T (C>T)	484/555	T allele reduced risk; TT genotype reduced survival pharyngeal cancer	HNSCC	Caucasian	[42]
**rs2292832**	**149**	**C>T**	**1852/1677**	**no association**	**HNSCC**	**Caucasian, Asian**	**[20]**
**rs2292832**	**149**	**C>T**	**1130/1109**	**no association**	**HNSCC**	**Caucasian, Asian**	**[25]**
**rs2292832**	**149**	**C>T**	**1031/2075**	**no association**	**OSCC**	**Asia**	**[28]**
rs2292832	149	(C>T)	1008/-	no association	OPSCC	Caucasian (Non-Hispanic white)	[33]
rs2292832	149	C (T>C)	470/425	no association; increased risk with betel nut chewing and tobacco use	OSCC	Asia (Taiwan)	[34]
rs2292832	149	(A>G)	576/1552	no association	HNSCC	Asia (Chinese)	[36]
rs2292832	149	(G>T)	1109/1130	no association	HNSCC	Caucasian (Non-Hispanic white)	[37]
rs2292832	149	CC (C>T)	996/-	reduced risk of death	SCCNOP	Caucasian (Non-Hispanic white)	[39]
rs2292832	149	CT, TT(C>T)	200/200	increased risk (susceptibility)	OSCC	Asia (Indian)	[43]
**rs3746444**	**499**	**A>G**	**1579/1555**	**no association**	**HNSCC**	**Caucasian, Asian**	**[20]**
**rs3746444**	**499**	**T>C**	**1130/1109**	**no association**	**HNSCC**	**Caucasian, Asian**	**[25]**
**rs3746444**	**499**	**G, GG (A>G)**	**965/969**	**increased risk**	**OSCC**	**Asia**	**[28]**
**rs3746444**	**499**	**C (T>C)**	**669/688**	**reduced risk in Caucasian population**	**HNSCC, NPC**	**Caucasian, Asian**	**[31]**
rs3746444	499	C (T>C)	340/340	increased risk (allele comparison)	OSCC	Asia (Chinese)	[32]
rs3746444	499	(T>C)	1008/-	no association	OPSCC	Caucasian (Non-Hispanic white)	[33]
rs3746444	499	C (T>C)	470/425	CC increased risk; increased risk with betel nut chewing and tobacco use	OSCC	Asia (Taiwan)	[34]
rs3746444	499	AG, GG (A>G)	1109/1130	reduced risk	HNSCC	Caucasian (Non-Hispanic white)	[37]
rs3746444	499	TT (T>C)	996/-	reduced risk of death	SCCNOP	Caucasian (Non-Hispanic white)	[39]
rs3746444	499	A>G	80/50	increased risk (susceptibility)	OSCC	Asia (Indian)	[43]
rs3746444	499	T>C	56/56	no association	OSCC	Asia (Iranian)	[44]
rs3746444	499	T>C	169 OL, 80 OSF, 155 OSCC/204-association susceptibility-BQ related OL, OSF, OSCC 512/668-susceptibility to OSCC	TC, CC increased risk of BQ-related OSF; TC, CC decreased risk of OSCC	OSCC	Asia (Taiwan)	[45]

1rs number is in the 1st column; the name of the miRNA is in the 2nd column; the change examined in the study and (if significant) the allele or genotype showing the effect is in the 3rd; the 4th column shows the ratio of the size of the study group to the control group; the 5th column shows the effect caused by change; the 6th column shows the type of cancer; the 7th column shows the ethnic group/population; and the 8th column shows the reference. Meta-analyses are highlighted in bold. Abbreviations: HNSCC—head and neck squamous cell carcinoma; NPC—nasopharyngeal carcinoma; OSCC—oral squamous cell carcinoma; PSCC—pharynx squamous cell carcinoma; OPSCC—oropharynx squamous cell carcinoma; SCCNOP—squamous cell carcinoma of the nasopharynx.

**Table 2:** Rare SNPs in miRNAs in HNSCC.

rs	miR	Allele/Genotype	Case/Controls	Role in HNC	Type of Cancer	Population	References
rs7210937	miR-1269b	CG, GG (G>C)	241 OPLs/188 APSCC/686	decreased risk among betel chewers	leukoplakia, OPL, HNSCC	Taiwanese	[35]
rs2043556	miR-605	AG, AA (A>G)	576/1552	decreased risk	OSCC	Chinese	[36]
rs4919510	miR-680	CG, GG (C>G)	906/1072	associated with the risk	NPC	Southern Chinese	[46]
rs10877887	let-7i	TC, CC (T>C)	3878/4725	decreased risk	HNC	Asian	[47]
rs578481	pri miR-101-1	G (A>G)	576/1552	associated with the risk	OSCC	Chinese	[48]
rs705509		A (G>A)					
rs7834169	548H4 upstream	C (G>C)	904/1051	associated with the risk	HNSCC	independent population	[49]
rs24168	miR-29a	AA (G>A)	299/452	decreased risk among betel chewers	leukoplakia	Indian	[50]
		AA, AG (G>A)	452/451	decreased risk among tobacco users	oral cancer	Indian	[51]
rs2187473	miR-34b	CT, TT (C>T)	299/452	decreased risk	leukoplakia	Indian	[50]
		CT, TT (C>T)	452/451	decreased risk	oral cancer	Indian	[51]
rs7372209	miR-26-a1	CT, TT (C>T)	136/136	associated with risk	OPL	White	[52]

rs number in the 1st column; the name of the miRNA in the 2nd column; the change examined in the study and (if significant) the allele or genotype showing the effect in the 3rd column; the ratio of the size of the study group to the control group in the 4th column; the effect caused by the change in the 5th column; the type of cancer in the 6th column; the ethnic group/population in the 7th column; and the reference in the 8th column. Abbreviations: HNSCC—head and neck squamous cell carcinoma; NPC—nasopharyngeal carcinoma; OSCC—oral squamous cell carcinoma; OPL—oral premalignant lesion; and HNC—head and neck cancer.

## Data Availability

Not applicable.

## Supplementary Materials

The following supporting information can be downloaded at https://www.mdpi.com/article/10.3390/genes15091226/s1, Table S1: Allele frequency of selected variants in populations according to dbSNP.
